# Stress effects on the top-down control of visuospatial attention: Evidence from cue-dependent alpha oscillations

**DOI:** 10.3758/s13415-022-00994-1

**Published:** 2022-04-05

**Authors:** Mauro F. Larra, Xinwei Zhang, Johannes B. Finke, Hartmut Schächinger, Edmund Wascher, Stefan Arnau

**Affiliations:** 1 Leibniz Research Centre for Working Environment and Human Factors, Ardeystr. 67, 44139 Dortmund, Germany; 2 Division of Clinical Psychophysiology, Institute of Psychobiology, University of Trier, 54290 Trier, Germany; 3 Department of Clinical Psychology and Psychotherapy, University of Siegen, 57076 Siegen, Germany

**Keywords:** Cortisol, Acute stress, EEG, Alpha, Executive functions, Information processing, Cold pressor test

## Abstract

Stress is assumed to inhibit the top-down control of attention and to facilitate bottom-up processing. Evidence from human experiments, however, remains scarce. Previous studies have addressed how stress affects the interplay of bottom-up and top-down mechanisms of attention. A key open question is in how far such effects can actually be attributed to a stress-induced modulation of top-down attention control. We sought to isolate top-down from bottom-up effects by assessing stress effects on anticipatory changes in alpha oscillations that precede stimulus processing. Participants performed in a cued target detection task in which a cue prompted them to covertly shift their attention to left or right screen positions, 20 min after being exposed to the bilateral feet cold pressor test or a warm water control procedure. The stressor led to a substantial increase in cortisol, peaking 20 min post stressor, along with rises in heart rate, blood pressure, and subjective ratings of stress and arousal. As expected, cued attention deployment led to higher alpha power over posterior electrodes contralateral versus ipsilateral to the attended hemifield during the cue-target interval. Importantly, this purely endogenous effect was potentiated by stress, however, significant differences were restricted to the middle of the cue-target interval and thus temporally separated from the appearance of the target. These results indicate that stress does not impair top-down attentional control per se but may introduce a qualitative change modulating the way attention is deployed to meet action goals.

## Introduction

The top-down control of selective attention allows us to filter out aspects relevant to our current goals from a continuous stream of information. According to a widely accepted model, this is achieved by modulating the cortical sensitivity to incoming stimuli. Specifically, top-down signals originating from a frontoparietal attention network bias neural competition in sensory areas that is otherwise driven by exogenous factors (i.e., salience) in favor of attended aspects (Corbetta & Shulman, 2002; Desimone & Duncan, 1995; Kastner & Ungerleider, 2000). For example, humans are able to orient their attention to a specific location in the visual field without moving their eyes, which results in an enhanced detectability for stimuli presented at the attended location at the expense of events occurring at unattended locations (Posner, 1980; Posner & Petersen, 1990). Such covert shifts of visuospatial attention go along with specific, spatially dependent and retinotopically organized modulations of neural activity in early visual areas. Single-cell recordings in monkeys demonstrate an increase in the firing rate of neurons receptive to the attended location even in the absence of visual stimulation (Fries et al., 2001; Luck et al., 1997; Motter, 1993). In humans, such a spatially specific anticipatory activation of visual sensory areas commensurate with the attended location has been demonstrated in studies measuring fMRI and oscillatory brain activity (Capotosto et al., 2009; Kastner & Ungerleider, 2001; Kastner et al., 1999; Thut et al., 2006; Worden et al., 2000).

Stress is assumed to inhibit the top-down control of attention, inducing a switch to a bottom-up, salience-based processing (Arnsten, 2015; Hermans et al., 2014). This effect has been directly linked to neuroendocrine changes under stress and their effect on central nervous processes. In particular, stress is characterized by an activation of the sympathetic nervous and adrenomedullary systems and the hypothalamus–pituitary–adrenal axis (HPA). This response cascade leads to a fast increase in central and peripheral catecholamines which is followed by a delayed rise in circulating cortisol owed to stepwise activation of the HPA (Ulrich-Lai & Herman, 2009). Animal studies have shown that stress levels of central noradrenaline inhibit activity within the prefrontal cortex (PFC) by acting on lower-affinity alpha- and beta-adrenergic receptors (Arnsten, 2009, 2015). Circulating cortisol readily enters the brain to act on mineral- and glucocorticoid membrane receptors further potentiating these down-regulatory catecholaminergic effects via a fast non-genomic mechanism (Barsegyan et al., 2010; de Kloet et al., 2008; Haller et al., 2008). Thus, stress-induced molecular changes impair PFC signaling and may thereby disrupt the neural basis for endogenous attentional control. At the same time, facilitatory effects of catecholamines and cortisol have been observed in subcortical and limbic structures such as the amygdala (Roozendaal et al., 2006; Wichmann et al., 2012). It therefore has been suggested that stress potentiates exogenous attention by activating these key structures of the salience network (Hermans et al., 2011). Consequently, stress may affect attention both by impairing top-down attentional control and by increasing stimulus-driven bottom-up processes.

Indeed, human neuroimaging studies demonstrate that stress inhibits higher-order cognition and related activity within the dorsolateral PFC (for review see Hermans et al., 2014; Joels et al., 2018). Moreover, stress has been shown to boost early stimulus evoked brain potentials and to attenuate later ones, indicating increased bottom-up processing (Elling et al., 2012; Sanger et al., 2014; Shackman et al., 2011). However, a key open question is in how far the effects of stress on top-down control of attention depend on concurrent bottom-up processing. To date, studies have investigated the influence of stress in situations that require attending to a target stimulus against other interfering stimuli. But results have been mixed reporting both improved (Kan et al., 2020; Qi & Gao, 2020; Schwabe & Wolf, 2010) and disrupted attention control (Plessow et al., 2011; Sänger et al., 2014). Interestingly, facilitatory effects of stress have been consistently observed in the attentional blink paradigm (Kan et al., 2019, 2020; Schwabe & Wolf, 2010), in which interfering stimuli are presented *before* the target stimulus appears. By contrast stress seems to disrupt attentional selection with simultaneously presented distractors (Sanger et al., 2014), hinting at a decisive role for concurrent bottom-up processes. Nevertheless, all post-stimulus phenomena necessarily reflect the result of an interaction between bottom-up and top-down mechanisms of attention. Inferring attentional effects based on differences in stimulus processing does therefore not allow to pinpoint these on a modulation of top-down control. However, quantifying stress effects on controlled anticipatory brain activity occurring before stimulus presentation may provide for such a differentiation.

The purpose of the present study was to isolate effects of stress on the top-down control of attention from concurrent bottom-up effects by directly examining oscillatory brain activity during visuospatial attention deployment preceding target processing. As described above, such endogenous anticipatory shifts of visuospatial attention lead to enhanced excitability of cortical areas responsive to the attended location. This phenomenon can be measured using the EEG by assessing alpha power at posterior recording sites, contralateral to the attended location, as relative decreases in alpha power index higher cortical activity (Capotosto et al., 2009; Sauseng et al., 2005; Thut et al., 2006; Worden et al., 2000). We used a variant of the Posner cueing task in which an auditory cue prompted participants to covertly direct their attention to either the left or right hemifield without moving their eyes. Stress exposure was manipulated in a within-subjects design: The participants underwent both a fully automated bilateral feet version of the Cold Pressor Test (CPT) and a warm-water control procedure on two separate dates, one week apart. Cortisol, cardiovascular reactions and subjective ratings were assessed to quantify stress responses. Following previous publications reporting significant attentional effects, attention was probed 20 min after the stressor, when cortisol responses can be expected to peak.

## Methods

### Sample

An a priori power analysis—conducted with G-power (Faul et al., 2007)—indicated a required sample size of 21 to detect a small-to-moderate within-subjects effect (*η*^*2*^ = 0.15) with 90% power.

Twenty-four, healthy, male participants were recruited via newsletter at the University of Trier and tested twice: one time under control and one time under stress conditions. The sequence of exposure to stress and control condition was counterbalanced. Participation was limited to right-handed men with normal weight (BMI between 19 and 25) and age between 18 and 35 years. Individuals were not included if they showed any evidence of acute or chronic diseases of the circulatory system (deviations from sine rhythm, glaucoma, Raynaud's disease, history of fainting, resting blood pressure above 140/90 mmHg), history of psychiatric disease or family history of arterial hypertension, and cerebral or aortic aneurisms. Furthermore, the following exclusion criteria were applied: regular smoking (> 5 cigarettes per day), drug intake or current use of medication except the occasional use of pain killers (paracetamol, acetylsalicylic acid, NSAIDs), increased objective or subjective sensitivity to cold, dermatologic lesions, burns or infections of the feet. Finally, participants were instructed to refrain from drinking alcohol for 24 h, caffeine for 12 h before the study, and omit vigorous exercise in the morning. A personal screening interview before participation determined whether all criteria for inclusion in the study were met.

All participants provided written, informed consent and were informed about their right to stop the experiment at any time. They were compensated with 80.00 € after completion of the study. All procedures were approved by the ethical committee of the state’s medical association (Landesärztekammer Rheinland-Pfalz) and were in accordance with the Declaration of Helsinki.

### Procedure

#### General Procedure

Experiments were conducted on 2 days separated by a 1-week interval. Each session was run between 1 and 5 pm to control for the diurnal cortisol cycle. A screening interview was conducted before the first session to assess exclusion criteria and brief participants about the procedure. They were informed that the experiment would include different physiological recordings assessed during a computerized attention task and several resting phases as well as a feet cold or warm water bath on both days. Participants were neither aware of which condition (cold vs. warm water) they would be subjected to on a particular date nor that conditions would alternate between sessions. Before the start of the actual experiment participants on both days performed a training and target titration session of the cueing paradigm (see below). Apart from the water temperature (CPT or warm water control condition), the study protocol was exactly the same for both days. Participants were sitting comfortably in a dimly lit stimulation chamber, completely isolated from noise and light artifacts. After preparation of electrodes and cuffs, participants were asked to put their bare feet into two special, separately fixed, 10-L tubs, which were still empty at this time. The experiment then started with a saliva sample followed by a 10-min resting period, during which heart rate and blood pressure were measured. Hereafter, participants provided another saliva sample, rated their current subjective stress and arousal levels, and were then informed that the water would now start to flow into the tubs. Depending on experimental condition, this was either ice-cold (stress condition) or warm water (control condition). After 3 min of CPT/control procedure, participants rated their current subjective stress and arousal levels. After that they were offered a towel to dry their feet. Another 10-min resting phase followed. After the end of the resting phase, the blood pressure cuff was removed and another saliva sample was taken. Then, a chinrest was mounted and adjusted so that participants could comfortably perform the following attention task. The cueing paradigm started 20 min after the CPT or control procedure and lasted for 45 min. Saliva samples were provided before the task after each block in 15-min intervals. After completion of the cueing paradigm, electrodes were removed and participants were dismissed.

#### Stress induction: Fully automated bilateral feet CPT

A fully automated bilateral feet version of the CPT (Bachmann et al., 2018; Larra et al., 2015) was conducted. Influx and efflux of the water were driven by hydrostatic pressure and regulated automatically via 14 valves with filling and draining times of less than 20 s, respectively. Controlling and timing of the valves was realized with LabVIEW software (National Instruments, Munich, Germany). Before starting the test, cold or warm water was stored in separate tanks with a capacity of 42 L each. The cold water tank (2–3 °C) was filled with tap water and crushed ice in fixed proportions; warm water temperature (36–37 °C) was set by tap water running through a flow-type heater. To avoid the formation of stable temperature layers next to the skin, the water around the feet was floating permanently at a flow rate of 0.2 m/sec. The cold and warm water exposure lasted for 3 min.

#### Cueing paradigm

**Fig. 1:**
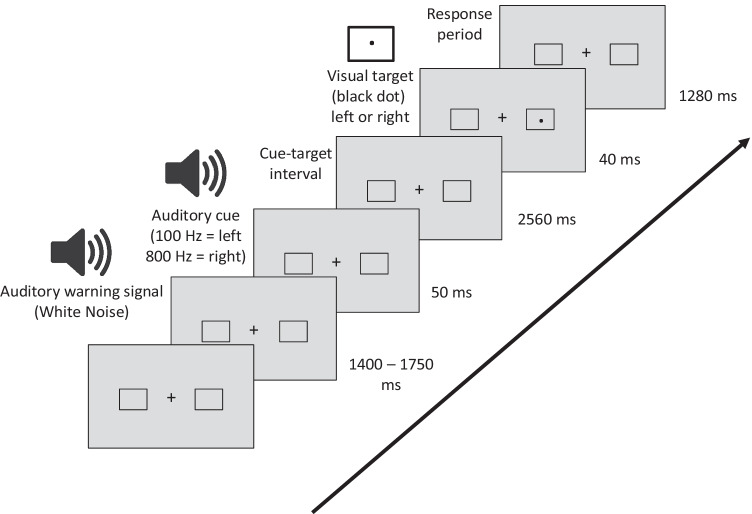
Sequence of events during a trial of the cueing paradigm

A variant of the endogenous Posner cueing task was employed, with task parameters adapted from Thut et al. (2006). Figure 1 illustrates the experimental paradigm. Two gray squares (3 * 3° visual angle) serving as position markers were constantly displayed left and right to the fixation cross (0.5°). Participants were asked to fixate on the central cross and to avoid eye movements and saccades. Stable viewing distance was supported by a head and chin rest and eye movements were monitored by electrooculogram (EOG). The start of a trial was indicated by an auditory warning signal (white noise) of variable length (1,400–1,750 ms), prompting participants to discontinue performance in the previous trial, resume to baseline position (attention on central cross), and to prepare for the upcoming cue. The warning signal was followed by a brief auditory cue (sine-wave tone, 50 ms) of either 100 or 800 Hz (randomly varied) prompting participants to covertly orient leftward or rightward, respectively, and to maintain attention at the corresponding position marker in the left or right visual field. After a delay of 2,560 ms, the target stimulus (black dot, presented at perithreshold size) was flashed for 40 ms in the center of one position marker (8° vertical and 26.5° horizontal eccentricity from the central fixation cross). Participants were asked to report detection of the targets on both the attended and unattended side via keyboard, using the right index finger for left targets and the right ring finger for right targets. Before testing, participants were explicitly told that targets would be presented at perithreshold size so that the detection task was going to be difficult. They were instructed to respond only when they actually perceived a target and to withhold responses or guesses if they failed to detect the target.

Before testing, participants completed a training session to familiarize themselves with the lab environment and the task, especially the concept of shifting spatial attention independently of eye movements. This also served to determine the individual perithreshold target sizes for each participant via target titration in three runs of 90 trials each during which five different target sizes were presented. Target sizes (in pixels) were 2*2, 2*3, 3*3, 3*4, and 4*4 (pixel size: 0.05 * 0.05°; longer axis horizontal for rectangular targets). After each run, performance feedback was displayed onscreen. Two adequate perithreshold targets (T1, T2) of adjacent sizes were selected for presentation during the experimental sessions (mean size T1: 2.3 * 2.8, T2: 2.8 * 3.3 pixels). During the experimental session, participants performed the target detection task in a total of 288 trials. Targets appeared at uncued positions in one third of the trials (catch trials), and participants were required to detect targets irrespective of the cued location. Performance was interspersed with regular 2-min breaks, during which saliva samples were obtained.

### Stress assessment

#### Cortisol

Saliva was collected using Salivettes (Saarstedt; Nümbrecht, Germany) and was sampled at the beginning of the experiment, after the baseline resting phase before the CPT as well as 15, 20, 35, 50, and 65 min after the CPT (or control procedure). Samples were kept at room temperature until the end of the session and then stored at − 20 °C. After thawing for biochemical analysis, the fraction of free cortisol in saliva (salivary cortisol) was determined using a time-resolved immunoassay with fluorometric detection, as described in detail elsewhere (Dressendorfer et al., 1992). Intra- and inter-assay coefficients of variation were between 4–7% and 7–9%, respectively.

#### Cardiovascular measurements

Heart rate and blood pressure during the intervention were measured at 0.5 and 2.5 min after the start of the CPT (or control procedure). Baseline and postintervention values were obtained from three measurements taken in 4-min intervals during the resting period before and after the CPT (or control procedure).

#### Subjective Ratings

Before and after the intervention, subjective ratings of current arousal and stress levels were assessed. Participants could place the mouse cursor on a visual analogue scale displayed on the monitor in front of the subject. They were asked to rate how stressed and aroused they currently felt from 0 (“not at all stressed”/ “aroused”) to 100 (“extremely stressed”/ “aroused”).

### EEG recording and analysis

#### Signal acquisition and preprocessing

EEG was derived from 32 active Ag/AgCl electrodes distributed on the scalp according to the 10–20 system (Easycap GmbH, Herrsching, Germany). EEG data were continuously sampled at 1,000 Hz using a BrainAmp amplifier (Brain Products, Gilching, Germany). Impedances were constantly kept below 10 kΩ.

EEG signal processing was performed in Matlab 2018b (The MathWorks Inc., Natick, MA) using custom scripts incorporating functions of the EEGLab toolbox (Delorme & Makeig, 2004). The data were bandpass-filtered at 1 to 30 Hz before corrupted channels were identified and removed based on kurtosis and probability criteria. On average, 0.7 channels (SD = 0.94) were removed. Subsequently, data were re-referenced to common average reference, resampled at 200 Hz, and segmented into epochs ranging from –1,500 ms to 4,000 ms relative to the onset of the cue. After automatic detection and removal of epochs containing artifacts, an independent component analysis was performed. Independent components (ICs) representing artifacts were identified and removed using ICLabel (Pion-Tonachini et al., 2019) by retaining only ICs which were labeled in the Brain IC-category with at least 0.5. On average,10.63 ICs (SD = 3.34) were excluded. Again, corrupt epochs were rejected automatically. The remaining epochs were manually checked for horizontal eye movements and trials in which participants did not maintain fixation during the cue target interval were removed. Participants with less than 100 valid trials per session were removed from all analyses (N = 4). An average of 215.65 (SD = 42.19) segments per participant and session entered the analysis.

#### Calculation of cue-dependent spectral lateralization

A time–frequency decomposition was performed on the preprocessed data via complex Morlet wavelet convolution. The wavelets were defined as complex sine waves tapered by a Gaussian. A set of 50 wavelets was used with frequencies ranging from 2 to 20 Hz in linearly spaced steps. The widths of the corresponding tapering Gaussians were defined in a way that the resulting wavelets had a temporal resolution ranging from 400 to 100 ms at full-width at half-maximum (FWHM; Cohen, 2019). This corresponds to a FWHM ranging from 1.75 to 8.75 Hz in the frequency domain. Power estimates were extracted by squaring the absolute values of the complex convolution result. In order to remove edge artifacts originating from convolving the data, the segments were pruned to –500 ms to 3,200 ms, relative to the onset of the cue. Finally, the lateralization index (LI) (Haegens et al., 2011; Tune et al., 2018) was calculated in time–frequency space for each lateralized pair of electrodes, defined as the difference of contralateral and ipsilateral power relative to the sum of contralateral and ipsilateral power ($${pow}_{ipsi}-{pow}_{contra})/{(pow}_{contra}+{pow}_{ipsi}$$). Negative values indicate a higher power contralateral to the cued side, positive values indicate a higher power value at ipsilateral recording sites. Before testing, a temporal smoothing was applied using a 50 frames-wide moving average window.

### Statistical analyses

Statistical testing of lateralization in time–frequency space was performed on LI data averaged across the channel pairs P7-P8, P3-P4, and CP5-CP6 using cluster-based permutation tests (Maris & Oostenveld, 2007). For each data point, t-statistics were computed and a clustering algorithm identified clusters of neighboring data points associated with a t-value corresponding to *p* < 0.05. The test statistic for each identified cluster was computed as the summed t-values of all data-points included. Type I error was controlled for by evaluating this test statistic under a H0 distribution of maximum cluster-level statistics which has been determined in a randomization procedure with 1,000 iterations. In each of these iterations, the maximum cluster statistic was identified based on data with randomized factor level assignments. Subsequently, the actually observed test statistics were compared against this H0 distribution. Clusters which exhibited summed t-values with *p* < 0.05 were regarded as significant.

Stress and performance data were analyzed by mixed-model Analyses of variance (ANOVA) and followed up by t-tests where appropriate.

## Results

### Stress induction

#### Cortisol

**Fig. 2:**
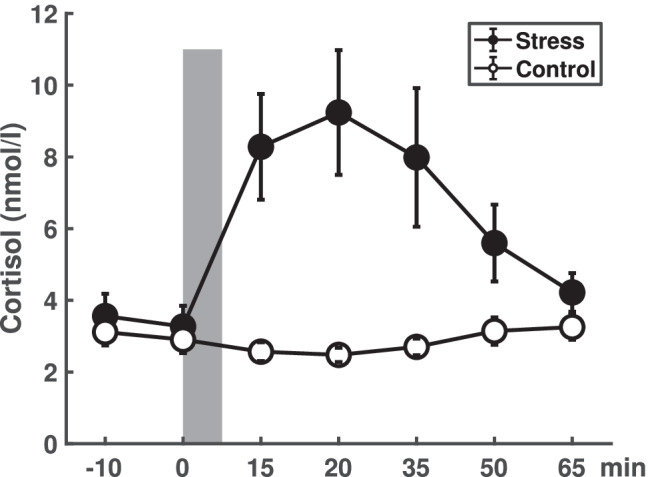
Depiction of mean cortisol profiles for the stress (filled circles) and control (empty circles) conditions. The grey bar indicates the timing of the CPT or control procedure. Error bars represent standard errors

The CPT successfully increased cortisol levels: A SEQUENCE (CPT first/control second vs. control first/CPT second) * CONDITION (CPT vs. control) * TIME (–10, 0, 15, 20, 35, 50, 65 min) mixed-model ANOVA on cortisol values revealed significant main effects of CONDITION (*F*[1, 18] = 10.871, *p* = 0.004, *η*^*2*^ = 0.37), TIME (*F*[6, 108] = 5.517, *p* = 0.007, *η*^*2*^ = 0.24) as well as a significant CONDITION * TIME interaction (*F*[6, 108] = 8.055, *p* < 0.001, *η*^*2*^ = 0.31). There was no difference in cortisol concentrations between stress and control condition at baseline (*t* < 1), whereas higher cortisol levels in the stress than in the control condition where evident until 50 min after the CPT (15 min: *t*[19] = 3.834, *p* < 0.001, *η*^*2*^ = 0.44; 20 min: *t*[19] = 3.918, *p* < 0.001, *η*^*2*^ = 0.45; 35 min: *t*[19] = 2.416, *p* = 0.013, *η*^*2*^ = 0.24; 50 min: *t*[19] = 1.868, *p* = 0.038, *η*^*2*^ = 0.16; 65 min: *t*[19] = 1.069, *p* = 0.15, *η*^*2*^ = 0.06; Fig. 2). These results were independent from the sequential order in which participants were exposed to either CPT or control condition as there were no significant main or interaction effects comprising SEQUENCE (all *F*-values < 1).

#### Cardiovascular measures

Blood pressure and heart rate were also increased by the CPT. Separate SEQUENCE * CONDITION * TIME (pre, during, post) within-subjects ANOVAs conducted on systolic (SYS), diastolic (DIA), mean arterial blood pressure (MAP) and heart rate (HR) values revealed significant main effects of TIME for SYS (*F*[2, 36] = 12.73, *p* < 0.001, *η*^*2*^ = 0.41), DIA (*F*[2, 36] = 13.898, *p* < 0.001, *η*^*2*^ = 0.44), MAP (*F*[2, 36] = 17.661, *p* < 0.001, *η*^*2*^ = 0.50), and HR (*F*[2, 36] = 19.412, *p* < 0.001, *η*^*2*^ = 0.5), as well as a significant interaction of CONDITION * TIME (SYS: *F*[2, 36] = 26.536, *p* < 0.001, *η*^*2*^ = 0.6; DIA: *F*[2, 36] = 21.089, *p* < 0.001, *η*^*2*^ = 0.54; MAP: *F*[2, 36] = 25.812, *p* < 0.001, *η*^*2*^ = 0.58; HR: *F*[2, 36] = 45.682, *p* < 0.001, *η*^*2*^ = 0.71). There was no difference in heart rate and blood pressure at baseline (SYS: *t*[19] = 0.35, *p* = 0.73; DIA: *t*[19] = 1.619, *p* = 0.122, *η*^*2*^ = 0.12; MAP: *t*[19] = 1.029, *p* = 0.317, *η*^*2*^ = 0.06; HR: *t*[*19]* = 1.003, *p* = 0.328, *η*^*2*^ = 0.05), but both were increased during the CPT compared with the control condition (SYS: *t*[19] = 4.943, *p* < 0.001, *η*^*2*^ = 0.65; DIA: *t*[19] = 3.019, *p* = 0.007, *η*^*2*^ = 0.32; MAP: *t*[19] = 4.106, *p* < 0.001, *η*^*2*^ = 0.47; HR: *t*[19] = 4.295, *p* < 0.001, *η*^*2*^ = 0.49). There was a significant interaction of SEQUENCE*CONDITION in SYS (*F*[1, 18] = 5.217, *p* = 0.035, *η*^*2*^ = 0.22), DIA (*F*[1, 18] = 8.8, *p* = 0.008, *η*^*2*^ = 0.33), MAP (*F*[1, 18] = 9.04, *p* = 0.008, *η*^*2*^ = 0.33) but not HR (*F* < 1), indicating marginally higher overall blood pressure values during the first day of the experiment, but there were no other significant interactions comprising SEQUENCE. Thus, stress responses were unaffected by sequential order of exposure. Mean values for cardiovascular responses and subjective ratings are shown in Table 1.

**Table 1:** Mean values and standard errors for cardiovascular parameters and subjective ratings before, during, and after the intervention in the CPT and control condition

	Pre	During	Post
SBP			
CPT	120.9 ± 2.5	135.1 ± 2.9	121.5 ± 2.1
control	121.8 ± 1.9	119.7 ± 2.3	119.4 ± 2.1
DBP			
CPT	65.9 ± 1.3	74.9 ± 1.4	66.7 ± 1.4
control	68.5 ± 1.4	66.9 ± 1.7	66.9 ± 1.4
MAP			
CPT	87.3 ± 1.3	96.5 ± 1.8	87.6 ± 1.1
control	88.5 ± 1.365	87.3 ± 1.4	87.1 ± 1.2
HR			
CPT	68.1 ± 2.7	79.1 ± 2.9	67.2 ± 2.6
control	70.1 ± 1.9	70 ± 2.1	72.3 ± 1.6
Stress rating			
CPT	17 ± 3.8	-	52.9 ± 5.8
control	17.2 ± 4.4	-	13.4 ± 4.4
Arousal rating			
CPT	14.2 ± 3.6	-	51.1 ± 7.3
control	18.2 ± 4.1	-	11.5 ± 3.6

#### Subjective ratings

The CPT led to an increase in subjective ratings of stress and arousal. Separate SEQUENCE * CONDITION * TIME (pre, post) within-subjects ANOVAs on stress and arousal ratings resulted in significant main effects of CONDITION (Stress: *F*[1, 18] = 22.124, *p* < 0.001, *η*^*2*^ = 0.55; Arousal: *F*[1, 18] = 9.582, *p* = 0.006, *η*^*2*^ = 0.35), TIME (Stress: *F*[1, 18] = 16.676, *p* < 0.001, *η*^*2*^ = 0.46; Arousal: *F*[1, 18] = 18.562, *p* < 0.001, *η*^*2*^ = 0.51) and a significant CONDITION*TIME interaction (Stress: *F*[1, 18] = 19.939, *p* < 0.001, *η*^*2*^ = 0.53; Arousal: *F*[1, 18] = 19.787, *p* < 0.001, *η*^*2*^ = 0.52). Stress and arousal ratings were significantly increased immediately after the CPT compared with the control condition values (Stress: *t*[19] = 5.597, *p* < 0.001, *η*^*2*^ = 0.62; Arousal: *t*[19] = 4.417, *p* < 0.001, *η*^*2*^ = 0.51), whereas there was no difference at baseline before the intervention (all *t*-values < 1*)*. Again, SEQUENCE was not a significant factor (all *F*-values < 1). Mean stress and arousal ratings are given in Table 1.

### Cueing paradigm

#### Behavioral data

**Fig. 3:**
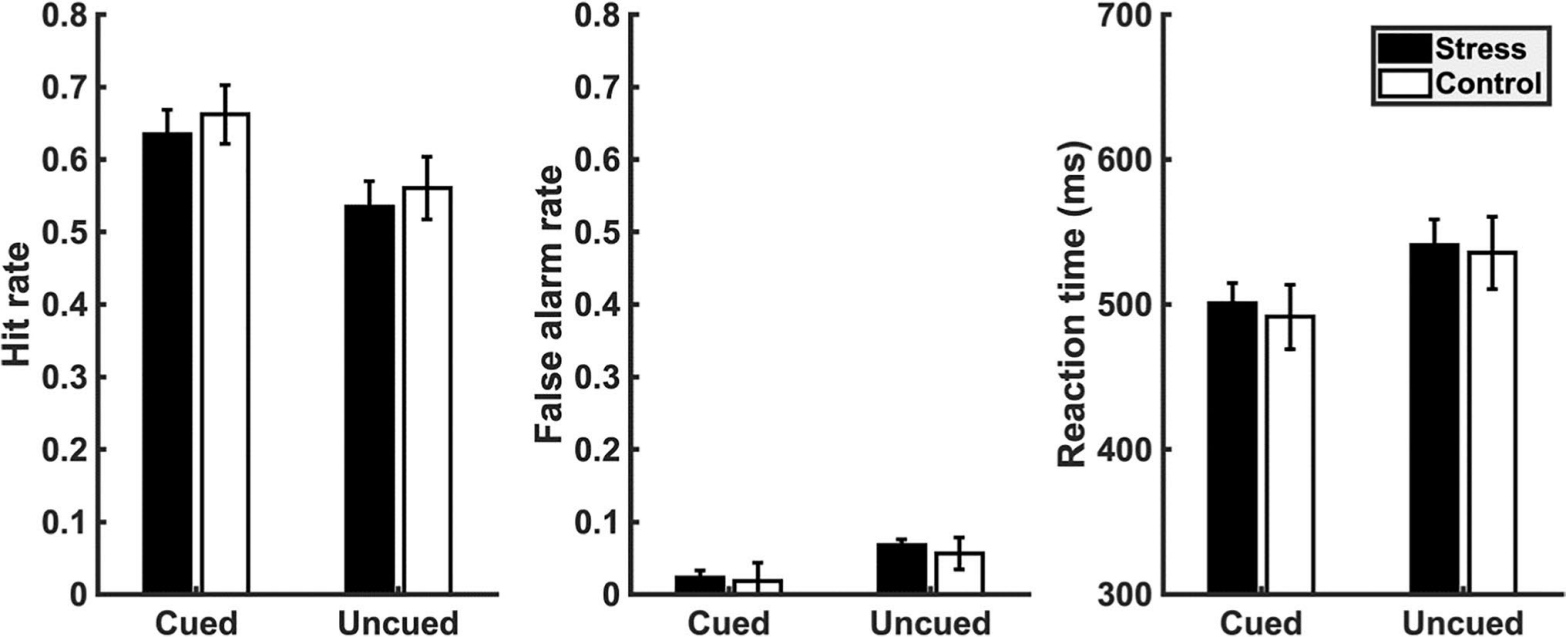
Mean values for hit rate (left panel), false-alarm rate (middle panel), and reaction time (right panel) for congruent (target at cued location) and incongruent (target at uncued location) trials within the stress (black bars) and control (white bars) conditions. Error bars represent standard errors

Cueing significantly affected both detection performance and latency (reaction times). Separate SEQUENCE * CONDITION * CUE (cued target vs. uncued target) mixed-model ANOVAs conducted on target detection rate (DR), false-alarm rate (FAR) and reaction times (RT) revealed a significant main effect of CUE for DR (*F*[1, 18] = 11.378, *p* = 0.003, *η*^*2*^ = 0.39) and RT (*F*[1, 18] = 8.469, *p* = 0.009, *η*^*2*^ = 0.32), indicating that the perithreshold targets were more likely to be perceived and detected earlier at cued than uncued positions (Fig. 3). Cueing also had a marginally significant effect on FAR (*F*[1, 18] = 4.488, *p* = 0.048, *η*^*2*^ = 0.2) with more false alarms when the target appeared at uncued positions. Importantly, however, FAR was still very low (0.06 ± 0.02 at uncued positions; Fig. 3), which shows that participants followed the instructions and did not just guess target appearance from cue location.

However, the effect of visuospatial attention deployment on performance was not modulated by stress as the critical interaction of CONDITION*CUE was far from being significant (HR, FAR and RT: *F* < 1). Also, there was no effect of stress on overall detection performance, because there were no main effects of CONDITION (HR and RT: *F* < 1; FAR: *F*[1, 18] = 1.318, *p* = 0.266, *η*^*2*^ = 0.06). Participants reacted faster on the second compared with the first day as evidenced by a SEQUENCE*CONDITION interaction effect for RT (*F*[1, 18] = 11.388, *p* = 0.003, *η*^*2*^ = 0.39) but not HR (*F*[1, 18] = 2.409, *p* = 0.138, *η*^*2*^ = 0.05) or FAR (*F* < 1). However, the effect of cueing remained unchanged between sessions as indicated by nonsignificant SEQUENCE*CONDITION*CUE interactions (HR and FAR: *F* < 1; RT: *F*[1, 18] = 2.481, *p* = 0.133, *η*^*2*^ = 0.12). Mean values and standard errors for HR, FAR and RT within the stress and control conditions are depicted in Fig. 3.

#### EEG data

**Fig. 4:**
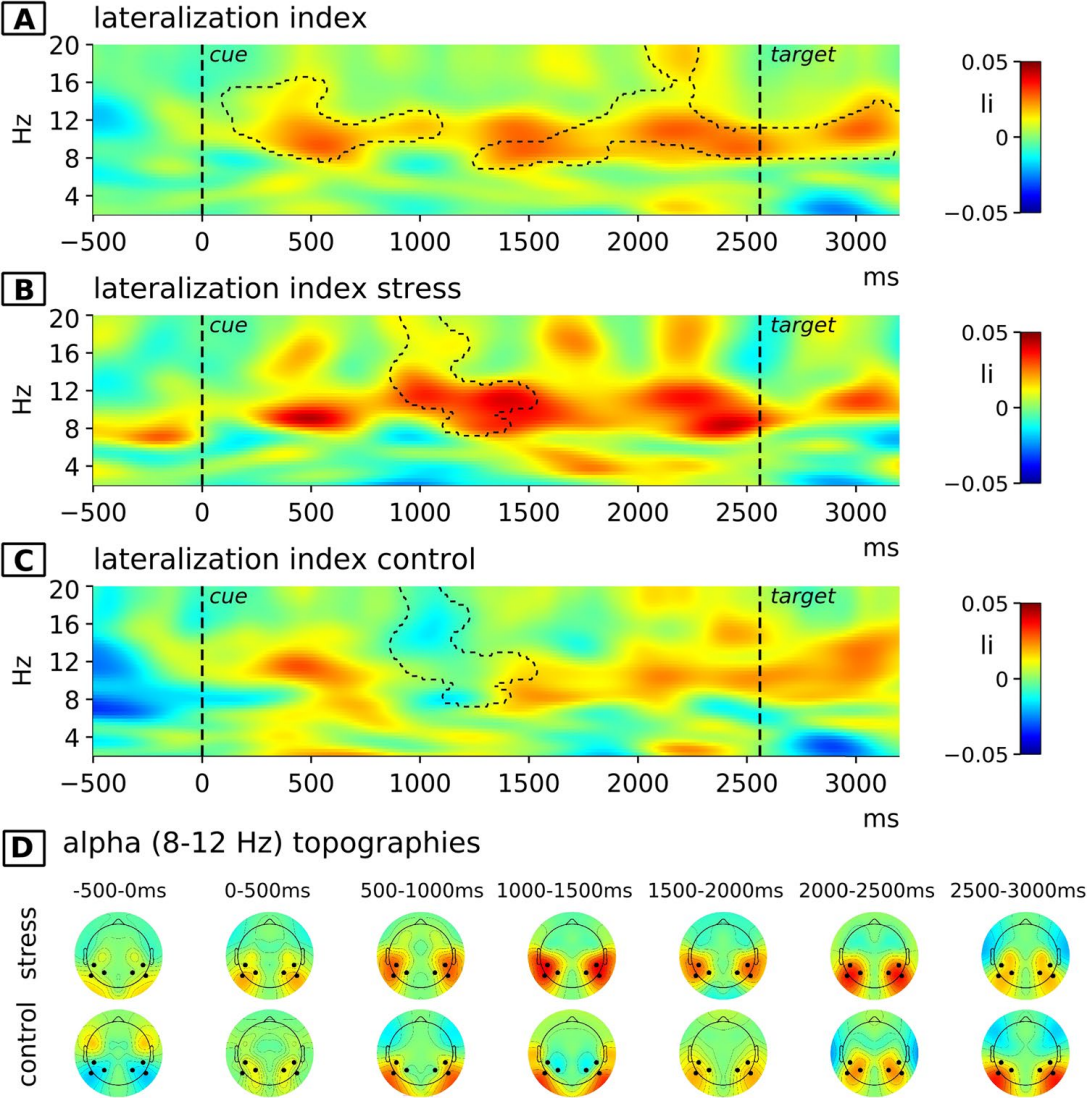
The lateralization index (LI) averaged across the channel pairs P7-P8, P3-P4, and CP5-CP6, as well as the results of the cluster-based permutation tests. (**A**) LI irrespective of the experimental condition. LI for the stress (**B**) and the control (**C**) conditions. Statistically significant clusters are indicated by dashed contour lines. The two clusters in **A** result from the test comparing the cue-dependent LI against null-hypothesis data, irrespective of the stress condition. The cluster illustrated in **B** and **C** is the same cluster, indicating the result from the test comparing the LI of the stress and the control condition. (**D**) Topographies of the LI in the alpha range at various time windows over the course of a trial for the stress and the control condition. Electrodes that were used for statistical analyses are highlighted. Because the LI is obtained for each pair of lateralized electrodes, the topographies are mirrored

Shifts in spatial attention in direction of the cue were also reflected in higher spectral power in the alpha range ipsilateral versus contralateral to the attended location during the cue-target interval (Fig. 4A). The statistical testing of electrophysiological lateralization in time–frequency space was performed by testing the lateralization index against null-hypothesis data generated by shuffling ipsi- and contralateral- electrodes on a single trial level. The cluster-based permutation test revealed two significant clusters, ranging from 95 to 1,100 ms and 1,250 to 3,200 ms relative to the onset of the cue in the temporal domain and from 7.88 to 16.33 Hz and 7.14 to 20 Hz in the frequency domain, respectively. Both clusters indicate a significant effect of the cue in terms of an increased lateralization of alpha band power during the cue-stimulus interval. To replicate previous research, we tested whether in addition significant alpha-lateralization was present within each condition alone by averaging the LI into three equally sized bins (0–800 ms, 800–1,600 ms, 1,600–2,400 ms) during the cue-target interval and testing it against 0. For the control condition, the results confirm a significant lateralization for the first (*t*[19] = 2.47, *p* = 0.023) and last (*t*[19] = 2.33, *p* = 0.031) but not for the middle bin (*t*[19] = 0.73, *p* = 0.475). In the stress condition, the lateralization was significant for all three bins (bin 1: *t*[19] = 2.34, *p* = 0.03; bin 2: *t*[19] = 3.71, *p* = 0.002, bin 3: *t*[19] = 3.15, *p* = 0.005).

In a next step, we directly compared the cue-dependent lateralization-indices of the stress and the control condition. For the corresponding cluster-based permutation test, the null hypothesis distribution was generated by permuting the labels of the stress versus control condition. The test revealed a significant cluster ranging from 870 to 1,535 ms and from 7.51 to 20 Hz (Figs. 4B, C). This result suggests a significantly stronger lateralization of alpha power in the stress compared with the control condition. Topographies of the alpha lateralization index over the course of a trial within the stress and control condition are depicted in Fig. 4D, across all recorded electrode sites for visualization purposes.

**Fig. 5:**
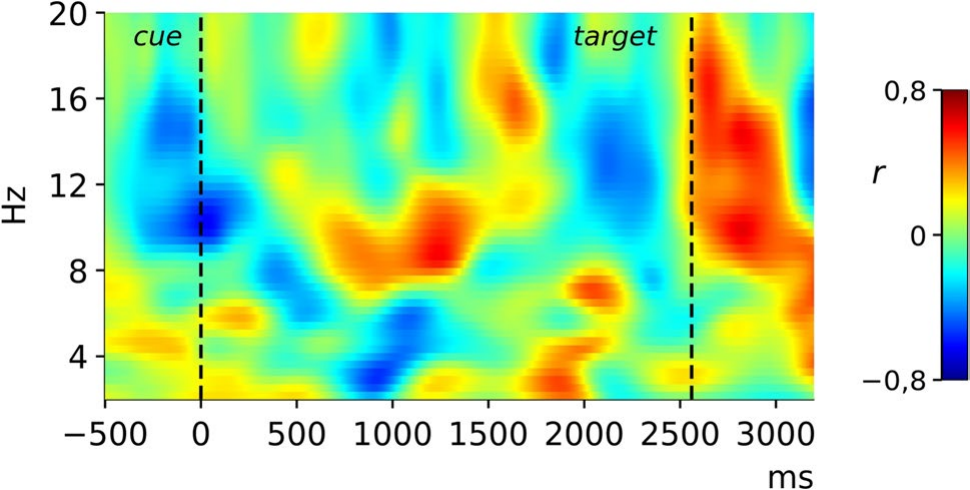
The pattern of correlations of the difference in the lateralization index between conditions (stress-control) and the difference of the cortisol parameter AUCi between conditions (stress-control)

To test whether the observed differences in alpha lateralization between the stress and the control condition were moderated by differences in cortisol levels, we calculated the correlation of intraindividual differences in the cortisol increase (area under the curve, AUCi; Pruessner et al., 2003) between sessions with the corresponding LI-differences (*Pearson’s r[Δli*_*stress-control*_*, ΔAUCi*_*stress-control*_*]*)*.* The results are depicted in Fig. 5 and indicate that the observed differences in alpha lateralization between the stress and control condition are positively correlated with cortisol-level differences. For an explorative analysis of these correlations in time × frequency space, again, a cluster-based permutation test was computed. As we computed the correlation of cortisol difference and differences in alpha lateralization, the null hypothesis distribution for this test is generated by permuting the *ΔAUCi* values between subjects, thereby breaking the systematic relation between *ΔAUCi* and *Δli*. No significant cluster could be observed in this test. However, the strongest correlations show some overlap with the effect observed when testing the alpha-lateralization of the stress versus the control condition regarding their position in time x frequency space. We therefore conducted an additional analysis to test whether the correlation values within this cluster significantly differed from the correlation during the rest of the cue-target interval. We averaged the correlations using Fisher’s Z-transformed values within the alpha-band for time points within this cluster (870–1,535 ms) as well as for time points from the rest of the cue-target interval. A dependent t-test comparing these averaged coefficients revealed a significant difference between correlations (*t*[2] = 1.88, *p* = 0.038), thus providing support for the specificity of correlations within the cluster in which stress and control conditions displayed significant differences in cue-dependent alpha lateralization.

## Discussion

The purpose of this study was to assess stress effects on the top-down control of spatial attention isolated from concurrent bottom-up mechanisms. We analyzed posterior alpha lateralization in the cue-target interval reflecting cue-dependent attentional top-down effects in the absence of stimulus processing. In line with previous findings (Capotosto et al., 2009; Rohenkohl & Nobre, 2011; Sauseng et al., 2005; Thut et al., 2006; Worden et al., 2000), we observed lower alpha power over posterior electrodes contralateral versus ipsilateral to the attended location suggesting enhanced activation of cortical areas responsive to the attended hemisphere. This effect set in approximately 100 ms after presentation of the cue, persisted throughout the entire cue-target interval, when no stimulation occurred, and therefore clearly reflects an endogenous mechanism.

Importantly, this effect was potentiated by stress: While descriptively, a stronger cue-dependent alpha lateralization after stress could be observed throughout almost the entire cue-target interval, the cluster permutation test revealed a significant cluster in the middle of the interval, when lateralization dropped in the control condition but was maintained in the stress condition. Moreover, the specificity of cortisol correlations observed within this interval also assigns relevance to the temporal location of the significant stress effects in alpha lateralization. It has been previously shown that apart from the spatial focus of attention also its temporal progression may be inferred from lateralized alpha (Foster et al., 2017; Rohenkohl & Nobre, 2011). Thus, a possible interpretation of our results may be that stress facilitates sustaining the spatial focus of attention over a longer period of time. This would, at first glance, speak against an impairing stress effect on PFC-dependent top-down control of attention, as has been proposed before (Arnsten, 2009, 2015). However, it should be noted that the temporal distance between cue and target was fixed. Therefore, it was possible to anticipate the moment when a target would be presented. Consequently, the drop in alpha lateralization within a timeframe irrelevant to task performance observed exclusively in the control condition may be the result of a more efficient deployment of attentional resources, based on the temporal expectation of the upcoming target. Such an interpretation also could account for the missing stress effect on task performance, because any benefits of an attentional boost may have been foiled by a failure to strategically adapt it according to task demands. In sum, these findings suggest that stress actually helps to focus visuospatial attention over a longer period of time. However, this seems to go along with a reduced capacity to flexibly adapt attentional control to a strategy for efficiently performing the task.

This is the first study to assess stress effects on the oscillatory signature of endogenous attention. In fact, to the best of our knowledge, only one published study has tested stress effects on attention within an endogenous cueing paradigm. In line with our findings, no behavioral effects of CPT induced stress could be observed; however, neither EEG nor cortisol data were acquired (Duecker et al., 2019). Other studies reporting stress effects on attentional processes employed a range of different conflict paradigms, such as the Stroop (Chajut & Algom, 2003; Kofman et al., 2006), Flanker (Qi & Gao, 2020) or Bar task (Sanger et al., 2014), but evidence has been mixed. Of particular relevance is the study by Sanger et al. (2014), since it also employed the CPT and probed stress effects on selective attention in the same timeframe with the help of EEG. The authors employed the bar task in which a luminance change of a stimulus had to be detected against a more salient but task irrelevant orientation change and found that stress impaired behavioral performance while evoked potentials (N1, N2pc) were indicative of reduced attention allocation to the relevant luminance change. While our results do not contradict the authors’ conclusion that stress induces a switch to a more bottom-up driven control of attention, they suggest that stress does not inhibit top-down control of attention per se, i.e., in the absence of concurrent bottom-up effects.

Alternatively, stress may directly affect bottom-up processing, e.g., by upregulating the salience network, as has been previously proposed (Hermans et al., 2014). Indeed, EEG studies inferring stress’ relative impact on top-down and bottom-up processes from the time-course of event related potentials unequivocally report a stress induced increase of early components in the N1 time-window indicating enhanced exogenous processing (Elling et al., 2011, 2012; Qi et al., 2018; Sanger et al., 2014; Shackman et al., 2011). Thus, our results may help to disambiguate the mixed pattern of findings concerning stress effects on attention by showing that previously observed effects in conflict tasks are unlikely to arise from an inhibition of endogenous attentional control per se but may rather depend on an interaction with or a direct effect on bottom-up processing as assessed in these studies.

A probable pathway by which stress could alter the processing of more complex attentional tasks would be an influence on PFC-dependent higher order executive processes. Rather than simply impairing endogenous attention, such an effect of stress could give rise to *qualitative* changes in top-down attention control. In fact, the difference in the temporal progression of alpha lateralization over the cue-target interval observed between stress and control conditions hints to such a rather specific effect on executive control. As outlined above, under control conditions participants seemed to engage in the task by rather strategically reducing the effort of focusing attention in the middle of the cue-target interval, when attentional focus was irrelevant to target perception. By contrast, the same participants when exposed to stress, although exhibiting a strong alpha-lateralization throughout the cue-target interval, failed to adjust this attentional boost according to task requirements. Importantly, a similar effect has recently reported by Kausche and Schwabe (2020), who found that stress increased EEG correlates of attention but abolished their specificity to predictive stimuli. Such an influence of stress also bodes well with a considerable body of research demonstrating an inhibition of strategical and target-oriented responding under stress. In particular, stress has been shown to promote habitual over instrumental behavior, an effect dependent on prefrontal and hippocampal mineralocorticoid receptors acting via a fast nongenomic mechanism (Schwabe & Wolf, 2009, 2011; Schwabe et al., 2012; Vogel et al., 2016). Indeed, the functional significance of differences in the middle of the cue-target interval is boldened by the specificity of correlations with differences in cortisol levels between conditions within this timeframe. Nevertheless, an alternative or additional explanation would be a stress-induced disruption of time perception, for which there also is some evidence (van Hedger et al., 2017). Thus, while fitting nicely into recent models of cortisol dependent stress effects on cognition, our interpretation regarding the differences in temporal evolution of cue-dependent alpha remains somewhat speculative. Further studies with a dedicated design are needed to disambiguate these possible interpretations.

A notorious difficulty in the reconciliation of findings within the stress literature is the difference in testing timeframes with respect to onset and offset of the stressor, because the stress response proceeds in several steps. An immediate and fast veining release adrenalin is followed by a delayed rise in circulating cortisol resulting from a stepwise activation of the HPA axis (Ulrich-Lai & Herman, 2009). Cortisol in turn acts on membrane and nuclear receptors to induce fast central nervous effects via nongenomic mechanisms as well as slower genomic effects (de Kloet et al., 2008). Adding further to complexity, stress induced changes via these different mechanisms are not always in the same direction but may be supportive, permissive, or even suppressive (Sapolsky et al., 2000). It is therefore important to point out that we tested stress effects after the peak in cortisol reactions, 20 min after stressor onset. Given the short duration of the CPT, at this time adrenergic effects can be expected to decrease, but cortisol starts to act via nongenomic mechanisms (Haller et al., 2008; Joels, 2018; Sapolsky et al., 2000). Recent models assume that early nongenomic effects of cortisol promote vigilance and simple behavioral strategies, while later genomic mechanism counteract these effects (Joels, 2018). Our results that demonstrate boosted endogenous attention along with a qualitative change in how it is controlled are compatible with this model and in line with previous reports of stress effects observed within the same timeframe, because they also demonstrate a qualitative change toward a simpler mode of attentional control (Kausche & Schwabe, 2020; Plessow et al., 2011; Sanger et al., 2014). However, comparisons with studies operating at different, particularly later, timeframes need to be drawn with care.

We employed a bilateral fully automated feet CPT and control procedure in each participant, providing maximal timing precision and standardization both between and within subjects. This also enabled us to directly assess stress induced changes within each participant, minimizing the impact of interindividual variability in stress responding and cue-dependent alpha-lateralization (Minami et al., 2020). Importantly, through bilateral exposure we could avoid unilateral somatosensory effects interfering with the spatial paradigm as would have been a potential limitation with the classic unilateral CPT. In line with previous reports (Bachmann et al., 2018; Larra et al., 2015), the stress induction procedure led to profound increases in subjective and cardiovascular parameters that were absent in the warm water control condition. Furthermore, the CPT induced a substantial cortisol reaction, whereas cortisol remained unchanged in the control condition. Thus, it can be concluded that the stress induction was successful and produced the intended reactions.

Nevertheless, stressor-specific characteristics should also be considered. As such, while the stress reaction is a stereotypic response, laboratory stressors may vary with respect to the orchestration of several stress response components (e.g., social evaluation particularly boosts cortisol reactions, Dickerson & Kemeny, 2004). Moreover, other widely used stress protocols, such as the Trier Social Stress Test (Kirschbaum et al., 1993), are cognitively demanding and have a far longer duration, whereas the CPT is characterized by physical aversiveness and a very short duration. These factors also may contribute to a change in brain state; therefore, different results may arise depending on the stress protocol used, even when testing timepoints relative to the onset of the stressor are comparable. Finally, our sample was restricted to healthy young men. However, stress effects have been found to vary with age and gender (Kudielka et al., 2004, 2009; Wolf, 2015), and further studies are needed to determine the extent to which our findings can be generalized across the entire population.

## Conclusions

We assessed stress effects on the top-down control of visuospatial attention independent from bottom-up effects by looking at cue-dependent changes in alpha activity that precede target processing. Our results demonstrate that, in a time-window characterized by high cortisol concentrations, stress rather facilitates than hampers these correlates of endogenous attention. However, stress also seems to alter the way how attention is deployed to meet task demands possibly by disfavoring a strategic approach. These results indicate that stress does not impair top-down attentional control per se but may rather affect higher order processes modulating the way attention is deployed to meet action goals. Tracing endogenous attentional processes via alpha oscillations is a promising method to dissect how exactly stress affects top-down control to alter information processing.

## Data Availability

Data and code underlying this manuscript can be accessed exclusively for scientific purposes on the Open Science Framework repository (https://osf.io/9wrea/), the code also at https://github.com/stefanarnau/stress-attention-EEG. The study was not preregistered.
